# Optimal Architecture of Floating-Point Arithmetic for Neural Network Training Processors

**DOI:** 10.3390/s22031230

**Published:** 2022-02-06

**Authors:** Muhammad Junaid, Saad Arslan, TaeGeon Lee, HyungWon Kim

**Affiliations:** 1 Department of Electronics, College of Electrical and Computer Engineering, Chungbuk National University, Cheongju 28644, Korea; junaid@chungbuk.ac.kr (M.J.); tglee2@chungbuk.ac.kr (T.L.); 2 Department of Electrical and Computer Engineering, COMSATS University Islamabad, Park Road, Tarlai Kalan, Islamabad 45550, Pakistan; saad.arslan@comsats.edu.pk

**Keywords:** floating-points, IEEE 754, convolutional neural network (CNN), MNIST dataset

## Abstract

The convergence of artificial intelligence (AI) is one of the critical technologies in the recent fourth industrial revolution. The AIoT (Artificial Intelligence Internet of Things) is expected to be a solution that aids rapid and secure data processing. While the success of AIoT demanded low-power neural network processors, most of the recent research has been focused on accelerator designs only for inference. The growing interest in self-supervised and semi-supervised learning now calls for processors offloading the training process in addition to the inference process. Incorporating training with high accuracy goals requires the use of floating-point operators. The higher precision floating-point arithmetic architectures in neural networks tend to consume a large area and energy. Consequently, an energy-efficient/compact accelerator is required. The proposed architecture incorporates training in 32 bits, 24 bits, 16 bits, and mixed precisions to find the optimal floating-point format for low power and smaller-sized edge device. The proposed accelerator engines have been verified on FPGA for both inference and training of the MNIST image dataset. The combination of 24-bit custom FP format with 16-bit Brain FP has achieved an accuracy of more than 93%. ASIC implementation of this optimized mixed-precision accelerator using TSMC 65nm reveals an active area of 1.036 × 1.036 mm^2^ and energy consumption of 4.445 µJ per training of one image. Compared with 32-bit architecture, the size and the energy are reduced by 4.7 and 3.91 times, respectively. Therefore, the CNN structure using floating-point numbers with an optimized data path will significantly contribute to developing the AIoT field that requires a small area, low energy, and high accuracy.

## 1. Introduction

The Internet of Things (IoT) is a core technology leading the fourth industrial revolution through the convergence and integration of various advanced technologies. Recently, the convergence of artificial intelligence (AI) is expected to be a solution that helps data processing in IoT quickly and safely. The development of AIoT (Artificial Intelligence Internet of Things), a combination of AI and IoT, is expected to improve and broaden IoT products’ performance [1,2,3]. 

AIoT is the latest research topic among AI semiconductors [4,5]. Before the AIoT topic, a wide range of research has been conducted in implementing AI, that is, a neural network that mimics human neurons. As research on AIoT is advancing, the challenges of resource-constrained IoT devices are also emerging. A survey on such challenges is being conducted by [6], where the potential solutions for challenges in communication overhead, convergence guarantee and energy reduction are summarized. Most of the studies on accelerators of the neural network have focused on the architecture and circuit structure in the forward direction that determines the accuracy of input data, such as image data [7,8]. However, to mimic the neural network of humans or animals as much as possible, it is necessary to implement a neural network circuit in the backpropagation that provides feedback through accuracy. Significant research is being conducted on studying the neural network circuit in the back direction [9,10]. Among the various applications of AIoT, wearable and smart home technologies have strict area and power constraints. Therefore, we need higher performance while consuming low power and size [11]. 

As explained in [12], learning in deployed neural networks can be of two types, which are on-chip learning [13,14] and off-chip learning [15,16]. Previously, high-speed servers were used to analyze the data, but several studies have concluded that it is more efficient to use edge devices to collect, process, and analyze data [17,18]. Edge devices that require repeated interactions with the server are prone to significant battery charge reduction. Minimizing the depreciation of battery power while interacting with the server is challenging. Kumar et al. [19] developed a tree-based algorithm for predicting resource-constrained IoT devices; still, they did not perform the training operation. Therefore, one of the key motivations in designing the optimized low power/area on-chip neural network is its ability to self-train its lightweight neural network model using various data inputs. For the realization of low power, it is essential to choose the appropriate format for the mathematical computations involved. The literature has shown that Integer operations are sufficient to design a low power inference accelerator [20,21]. However, considerable research is still required to train a neural network with reasonable accuracy as well. Empirical results from these studies suggest that at least 16-bit precision is required to train a neural network [22,23]. Mixed precision training using Integer operations is implemented in [22], which multiplies the two INT16 numbers and stores the output into an INT32 accumulator. One drawback mentioned by the authors in [22] was the lower dynamic range. The deficiency of Integer operators to represent a broad range of numbers served as the main obstacle to using them in training engines. The floating-point operators are used in [24] to train the weights to counter this problem. 

Floating-point operation demonstrates superior accuracy over fixed-point operation when employed in training neural networks [25,26]. Conventional neural network circuit design studies have been conducted using floating-point operations provided by GPUs or fixed-point computation hardware [27,28]. However, most of the existing floating-point-based neural networks are limited to inference operation, and only a few incorporate training engines that are aimed at high-speed servers, not low-power mobile devices. Since FP operations dissipate enormous power and consume a larger area to implement on-chip, the need to optimize the FP operation has emerged. One of the most used methods to reduce the complexity of the computation is the computing approximation technique, which can minimize significant energy consumption by FP operators [29]. While computing approximation has shown promising results concerning energy efficiency and throughput optimization, recent studies have focused on maintaining the precision of weight updates during backpropagation. In [30], mixed precision training is implemented by maintaining the master copy of FP32 weights, and another copy in FP16 is used during forward and backward pass. Then, the weight gradient updates the FP32 master copy and the process is repeated for each iteration. Although the overall memory usage was reduced, maintaining the copy of weights increased the memory requirement of weights by two times as well as the latency due to additional memory access. We came up with a novel technique to counter the shortcomings in precision reduction. An algorithm is designed to find the optimal floating format without sacrificing significant accuracy. Our method uses a mixed-precision architecture in which the layers requiring higher precision are assigned more bits, while those layers requiring less precision are assigned with lesser bits. 

This paper evaluates different floating-point formats and their combinations to implement FP operators, providing accurate results with less consumption of resources. We have implemented a circuit that infers accuracy using CNN (convolutional neural network) and a floating-point training circuit. We have used MNIST handwritten digit dataset for evaluation purposes. The prominent contributions of our paper are summarized below:

1. Designing optimized floating-point operators, i.e., Adder, Multiplier, and Divider, in different precisions.
2. Proposing two custom floating-point formats for evaluation purposes.
3. Designing an inference engine and a training engine of CNN to calculate the effect of precision on energy, area, and accuracy of CNN accelerator.
4. Designing a mixed-precision accelerator in which convolutional block is implemented in higher precision to obtain better results.

Section 2 describes the overview of different floating-point formats and the basic principles of CNN training architecture. Section 3 explains the proposed architecture of floating-point arithmetic operators and their usage in the CNN training accelerator. Section 4 focuses on the results of both FP operators individually and the whole CNN training accelerator with multiple configurations. Section 5 is reserved for the conclusion of this paper. 

## 2. Architecture CNN Training Accelerator

### 2.1. CNN Architecture

**Figure 1:**
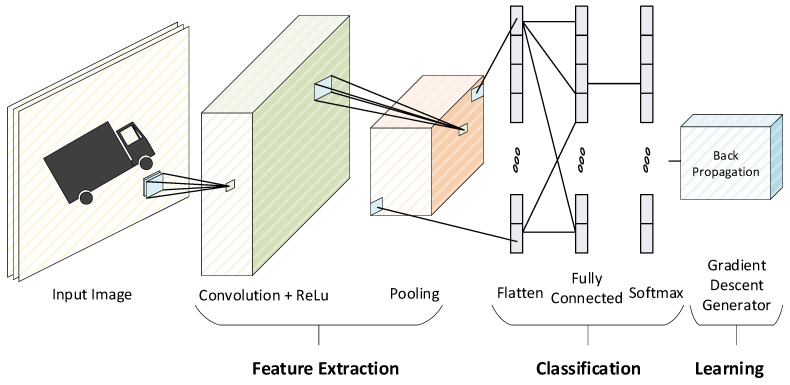
General architecture of CNN.

As we know, ANNs (artificial neural networks) resemble humans’ biological neural network operations. One of the distinguished ANN circuits is convolutional neural network (CNN), most commonly used for feature extraction and classification. A CNN training accelerator is used to train the circuit to classify input images efficiently and accurately. The CNN training accelerator’s architecture allows it to infer the output from the input value using trained weights during forward propagation and update the weights during backpropagation, increasing the overall accuracy for the next image in forward propagation. The general CNN architecture is shown in Figure 1.

#### 2.1.1. SoftMax Module

**Figure 2:**
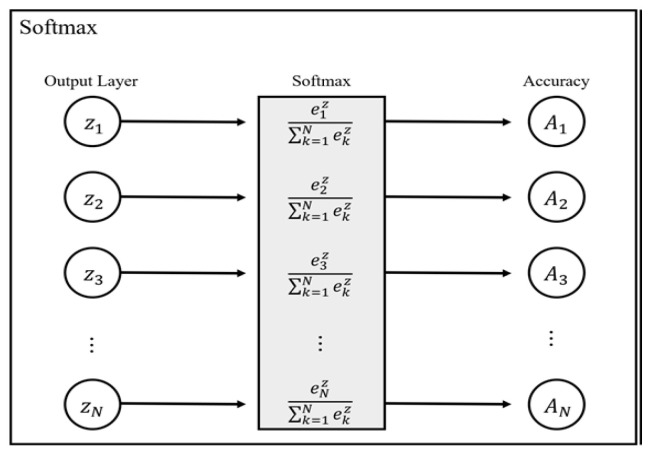
SoftMax Function.

As mentioned in [31], the SoftMax function can be described as a normalized exponential function. We have used it as an activation function to normalize the network’s output for output class based on probability distribution. The SoftMax function takes a vector z of N real numbers as input. It normalizes it to a probability distribution consisting of N probabilities proportional to the exponent of the input number. Before SoftMax is applied, some vector components may be negative or greater than 1, and the sum may not be 1. Using SoftMax, the sum of the elements becomes one, and each part is located between {(0, 1)} and can be interpreted as a probability. The SoftMax function can be expressed by Equation (1), and all input values are set to have values between 0 and 1 by applying a standard exponential function to each input vector. Then, after adding and accumulating all the exponent values, each input vector is normalized by dividing the sum of the accumulated exponents by each input vector exponent value.

(1) $$S{\left(x\right)}_{i^{\;}}=\frac{e^{x_{i}^{\;}}}{\sum_{i=1}^{M}e^{x_{i}^{\;}}}\;\;for\;i=1,2,\;\ldots M\;\&\;\;x=\left(x_{1^{\;}},\;x_{1^{\;}}\ldots \;x_{M}\right)\in Real\;number$$

is evident from Equation (1), a divider module and an adder module are needed to calculate the SoftMax value. Figure 2 shows the calculation process of the SoftMax function

In Figure 2, A_1_-A_N_ is the representation of the output layer’s values Z_1_-Z_N_ with a probability between 0 and 1.

#### 2.1.2. Gradient Descent Generator Module

Most of the deep neural network training models are still based on the backpropagation algorithm, which propagates the errors from the output layer backward and updates the variables layer by layer with the gradient descent-based optimization algorithms. Gradient descent plays an essential role in training the deep learning models, and lots of new variant algorithms have been proposed in recent years to improve its performance further. To smooth the fluctuation encountered in the learning process for the gradient descent, algorithms are proposed to accelerate the updating convergence of the variables.

Weights in our CNN module are updated using Equation (2).

(2) $$\theta^{\tau }=\theta^{\left(\tau -1\right)}-\eta *\;\Delta \nu^{\left(\tau \right)}$$

where $\theta^{\tau }$ is called moment at time τ, η is the learning rate, and $\Delta \nu^{\left(\tau \right)}$ is gradient. As shown in Equation (2), a multiplier and a subtractor module are needed to update the weights using gradient descent. 

## 3. Architecture of Floating-Point Arithmetic for CNN Training

### 3.1. General Floating-Point Number and Arithmetic

Three elements represent the floating-point format defined by the IEEE 754 standard [32]:

(1) Sign (Positive/Negative).
(2) Precision (Significant digit of real number, mantissa).
(3) Number of digits (Index range).

Floating points can be expressed as Equation (3)

(3) $$\mathrm{Floating}-\mathrm{point}\;\mathrm{Number}={\left(-1\right)}^{Sign}\cdot 1.M\cdot (2^{E-\left(exponent\;bias\right)})\;$$

Here, ‘*E*’ is the binary value of the exponent, and an exponent bias is the median value of the Exponent range and is used to indicate the 0 of an Exponent. Finally, ‘*M*’ is the mantissa, the part of the number after the decimal point. 

**Figure 3:**
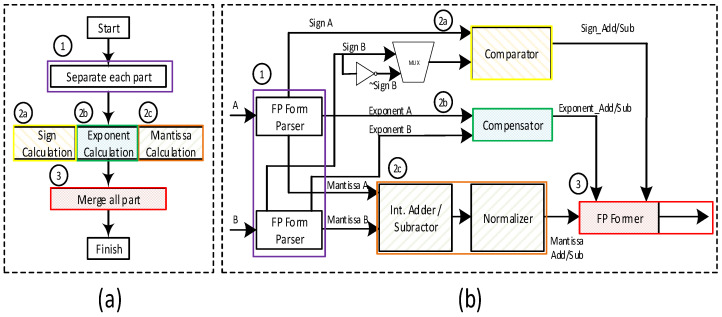
(**a**) General operation process. (**b**) Detailed operation process of floating point.

All floating-point operations follow the operations shown in Figure 3, where each part of the floating-point number is calculated separately. 

The steps for floating-point operations are summarized below:

1. Before performing the actual computation, original floating-point numbers A and B are partitioned into {sign A, exponent A, mantissa A} and {sign B, exponent B, mantissa B}.
2. For each separated element, perform a calculation suitable for the operation:
  - Sign: In addition/subtraction, the output sign is determined by comparing the mantissa and exponent of both inputs. A Not Gate and a Multiplexer are placed at the sign of input B to reverse the sign to use the same module for subtraction, while for multiplication/division, the sign of both inputs is calculated by XOR operation on the two input signs.
  - Exponent: In the case of difference in exponent values, the larger exponent value is selected among the two inputs. For the input with a smaller exponent, the mantissa bits are shifted towards the right to align the two numbers to the same decimal point. The difference between the two inputs’ exponent size determines the number of times the right shift to be performed.
  - Mantissa: this calculates the value of the Mantissa through an unsigned operation. There is a possibility that the result of the addition/subtraction operation for Mantissa bits becomes 1 bit larger than the Mantissa bit of both inputs. Therefore, to get precise results, we increased the size of Mantissa bits for both inputs twice and then performed the addition/subtraction of Mantissa based on the calculation results of Mantissa, whether MSB is 0 or 1. If the MSB is zero, a normalizer is not required. If MSB is 1, the normalizer moves the previously calculated Exponent bit and the Mantissa bit to obtain the final merged results.
3. Finally, each calculated element is combined into one in the floating-point former block to make a resultant floating-point output.

### 3.2. Variants of Floating-Point Number Formats

A total of four different floating-point formats have been evaluated and used to optimize our CNN. Table 1 shows the details for each of the formats.

**Figure 4:**
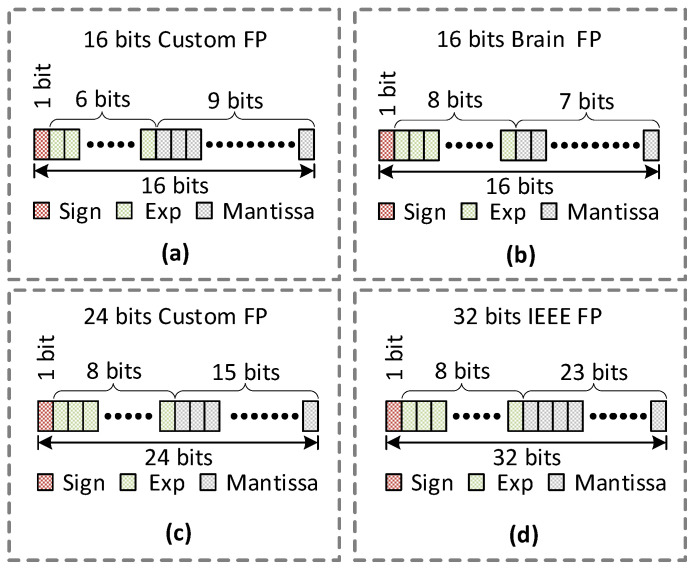
The representation of floating-points (**a**) 16-bit custom floating points; (**b**) 16-bit brain floating point; (**c**) 24-bit custom floating point; (**d**) 32-bit single precision.

Significand bits in Table 1 mean bits, including both Sign bits and Mantissa bits. Figure 4 represents each of the floating-point formats used in this paper.

The 16-bit custom floating-point format is proposed for comparison purposes with the existing 16-bit brain floating-point format. A 24-bit custom floating-point format is also presented for comparison of performance with other floating-point formats. We have also used this custom format for accumulation in a 16-bit convolution block to improve the accuracy of the network.

### 3.3. Division Calculation Using Reciprocal

There are many algorithms for accurate division calculations, but one of the most-used algorithms is the Newton–Raphson method. This method requires only subtraction and multiplication to calculate the reciprocal of a number. In numerical analysis, the real-valued function *f*(*y*) is approximated by a tangent line, whose equation is found from the value of *f*(*y*) and its first derivative at the initial approximation. If $\;y_{n}$ is the current estimate of the true root then the next estimate $y_{n+1}$ can be expressed simply as Equation (4).

(4) $$y_{n+1}=y_{n}-\frac{f\left(y_{n}\right)}{f^{\prime }\left(y_{n}\right)}$$

where $f^{\prime }\left(y_{n}\right)$ is the first derivative of $f\left(y_{n}\right)$ with respect to *y*. The form of Equation (4) is a form close to the recursive equation, and it is obtained through several iterations to obtain the desired reciprocal value. However, due to the recursive nature of the equation, we should not use the negative number. Since Mantissa bits used as significant figures are unsigned binary numbers, negative numbers are not used in our case. 

There is no problem in finding an approximation value for the initial value, no matter what value exists. However, the number of repetitions changes depending on the initial value, so we implemented the division module to obtain the correct reciprocal within six iterations.

**Figure 5:**
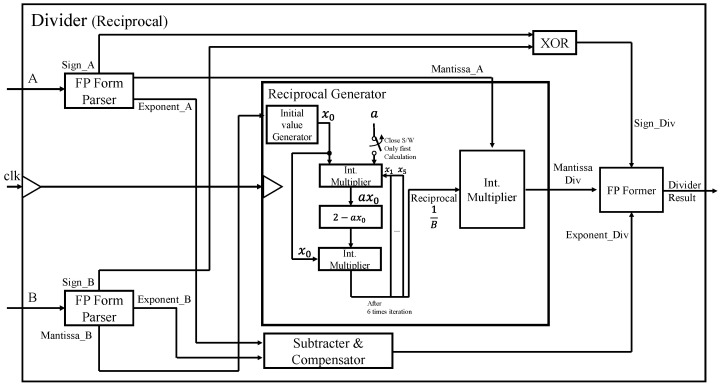
The architecture of floating-point divider using reciprocal.

As shown in Figure 5, we have three integer multipliers inside the reciprocal generator, out of which two multipliers are responsible for generating a reciprocal after multiple iterations. To perform the rapid calculations inside the reciprocal generator, we used the Dadda multiplier instead of the commonly used integer multiplier. The Dadda multiplier is a multiplier in which the partial products are summed in stages of the half and full adders. The final results are then added using conventional adders.

## 4. Proposed Architecture

### 4.1. Division Calculation Using Signed Array

**Figure 6:**
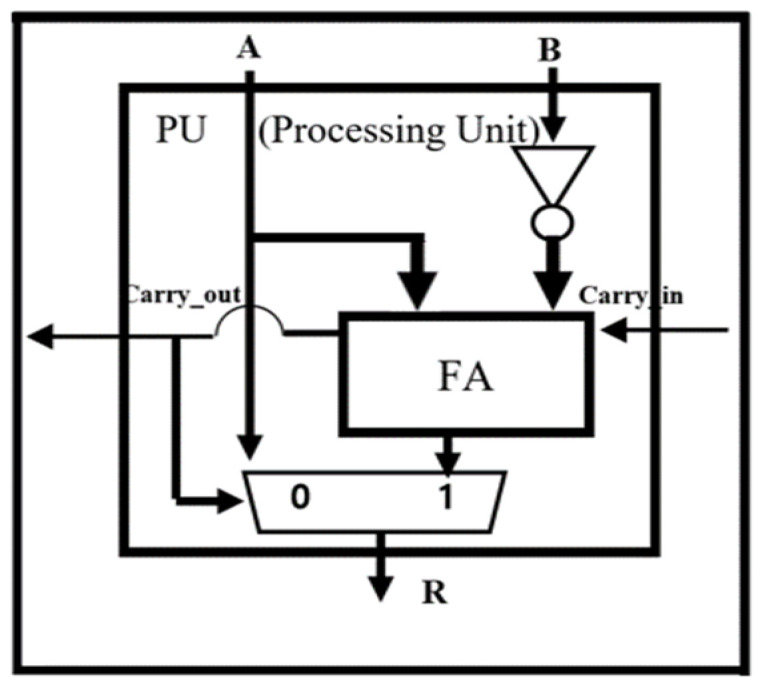
The Structure of processing unit for Signed Array division.

Since the reciprocal-based divider requires many iterations and multipliers, it suffers from long processing delay and an excessive hardware area overhead. We have proposed a division structure using a Signed Array to calculate a division calculation of binary numbers to counter these issues. Since Signed Array division does not have repetitive multiplication operations compared to division calculations using reciprocals, it offers significantly shorter processing delay than the reciprocal-based divider. It uses a specially designed Processing Unit (PU), as shown in Figure 6, optimized for division. It selects the quotient through the subtraction calculation and feedback of carry-out. 

**Figure 7:**
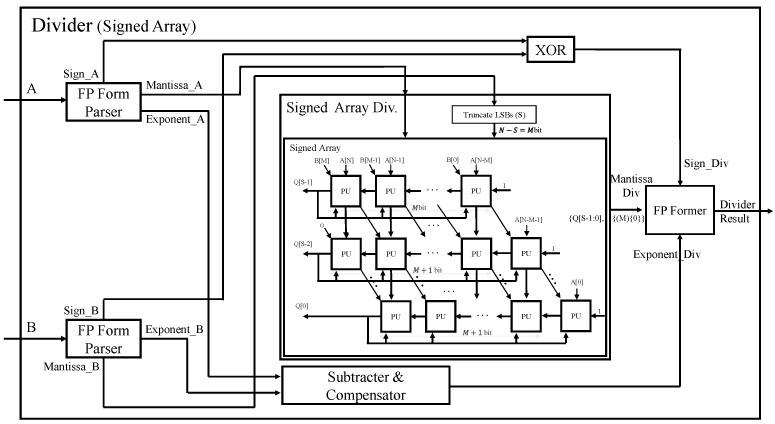
Structure of floating-point division operator using Signed Array.

The structure of the overall Signed Array division operator is shown in Figure 7. It first calculates the Mantissa in the signed array module, exponent using a subtractor and compensator block, and the sign bit of the result using XOR independently. Every row in the signed array module computes the partial division (subtracting from the previous partial division) and then passes it to the next row. Each row is shifted to the right by 1 bit to align each partial division corresponding to the next bit position of the dividend. Finally, just like the hand calculation of A divided B in binary numbers, each row of the array divider determines the next highest bit of quotient. Finally, it merges these three components to obtain the final divider result.

As emphasized in the introduction section, the architecture of our accelerator minimizes the energy and hardware size to the level that suffices the requirement for IoT applications. To compare the size, the operation delay, and the total consumption, we implemented the two dividers using a synthesis tool, Design Compiler, with TSMC 55 nm standard cells, which are analyzed in Table 2. 

The proposed Signed Array divider is 6.1 and 4.5 times smaller than the reciprocal-based divider for operation clocks of 50 MHz and 100 MHz, respectively. The operation delay of the Signed Array divider is 3.3 and 6 times shorter for the two clocks, respectively. Moreover, it significantly reduces the energy consumption by 17.5~22.5 times compared with the reciprocal-based divider. Therefore, we chose the proposed Signed Array divider in implementing the SoftMax function of the CNN training accelerator. 

### 4.2. Floating Point Multiplier

**Figure 8:**
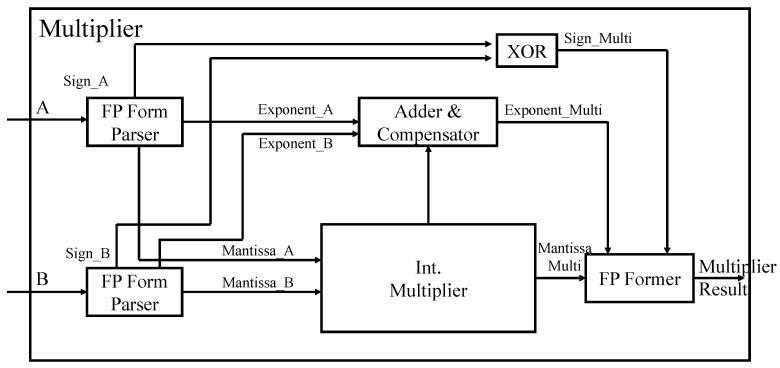
The architecture of proposed floating-point multiplier.

Unlike floating-point adder/subtracter, floating-point multipliers calculate Mantissa and exponent independent of the sign bit. The sign bit is calculated through a 2-input XOR gate. The adder and compensator block in Figure 8 calculates the resulting exponent by adding the exponents of the two input numbers and subtracting the offset ‘127’ from the result. However, if the calculated exponent result is not between 0 and 255, it is considered overflow/underflow and saturated to the bound as follows. Any value less than zero (underflow) is saturated to zero, while a value greater than 255 (overflow) is saturated to 255. The Mantissa output is calculated through integer multiplication of two input Mantissa. Finally, the Mantissa bits are rearranged using the Exponent value and then merged to produce the final floating-point format.

### 4.3. Overall Architecture of the Proposed CNN Accelerator

**Figure 9:**
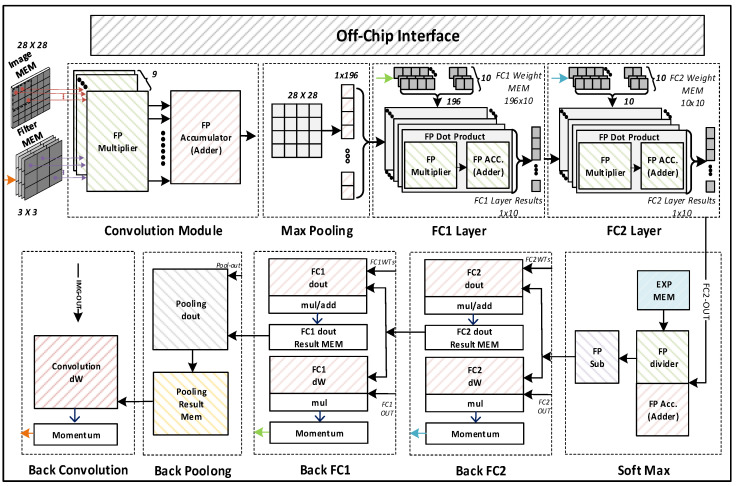
Overall architecture of the proposed CNN accelerator.

To evaluate the performance of the proposed floating-point operators, we have designed a CNN accelerator that supports two modes of operation, i.e., Inference and Training. Figure 9 shows the overall architecture of our accelerator with an off-chip interface used for sending the images, filters, weights, and control signals from outside of the chip.

Before sending the MNIST training images with size 28 × 28, the initial weights of four filters of size 3 × 3, FC1 with size 196 × 10, along with FC2 with size 10 × 10 are written in the on-chip memory through an off-chip interface. Upon receiving the start signal, the training image and the filter weights are passed to the convolution module, and the convolution is calculated based on the dot product of a matrix. The Max Pooling module down-samples the output of the convolution module by selecting the maximum value in every 2 × 2 subarray of the output feature data. Then, the two fully connected layers, FC1 and FC2, followed by the Softmax operation, predict the classification of the input image as the inference result.

During training mode, the backpropagation calculates gradient descent in matrix dot products in the reverse order of the CNN layers. Softmax and backpropagation layers are also involved to further train the partially trained weights. As evident from Figure 9, fully connected layers are divided into two modules, i.e., dout and dW. The dout module is used to calculate the gradient, while the dW module is used to calculate weight derivatives. Since back convolution is the final layer, there is therefore no dout module for this block. In this way, we train the weight values for each layer by calculating dout and dW repeatedly until the weight values achieve the desired accuracy. Those trained weights are stored in the respective memories which are used for inference in the next iteration of the training process. 

### 4.4. CNN Structure Optimization

This section explains how we optimize the CNN architecture by finding the optimal floating-point format. We initially calculated the Training/Test accuracies along with the dynamic power of representative precision format to find the starting point with reasonable accuracies, as shown in Table 3.

For our evaluation purposes, we chose the target accuracy in this paper of 93%. Although the custom 24-bit format satisfies the accuracy threshold of 93%, it incurs dynamic power of 30 mW, which is 58% higher than the IEEE-16 floating-point format. Therefore, we developed an algorithm that searches optimal floating-point formats of individual layers to achieve minimal power consumption while satisfying the target accuracy.

**Figure 10:**
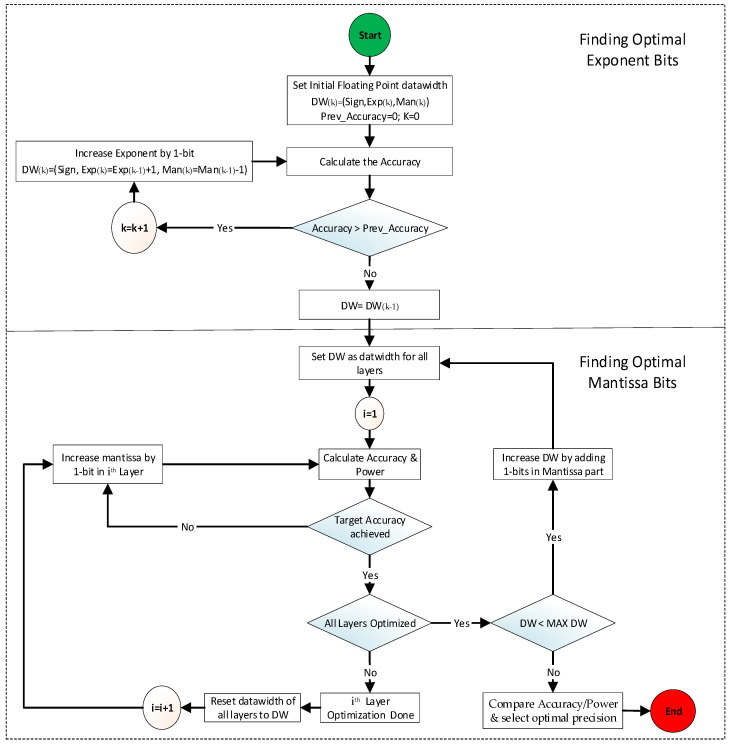
Arithmetic optimization algorithm.

The algorithm shown in Figure 10 first calculates the accuracy using the initial floating-point format which is set to IEEE-16 in this paper, and using Equation (5), it gradually increases the exponent by 1 bit until the accuracy stops increasing or starts decreasing.

(5) $$DW_{\left(k\right)}=(Sign,\;Exp_{\left(k\right)}=Exp_{\left(k-1\right)}+1,\;\;Man_{\left(k\right)}=Man_{\left(k-1\right)}-1)$$

As shown in Equation (5), the exponent bit in the k^th^-iteration is increased while the overall data width (DW) remains constant as the Mantissa bit is consequently decreased. After fixing the exponent bit width, the algorithm calculates the performance metric (accuracy and power) using the new floating-point data format. In the experiment of this paper, the new floating-point format before Mantissa optimization was found to be (Sign, Exp, DW-Exp-1) with DW of 16 bits, Exp = 8, and Mantissa = 16 – 8 – 1 = 7 bits. Then, the algorithm optimizes each layer’s precision format by gradually increasing the Mantissa by 1 bit until the target accuracy is met using the current DW. When all layers are optimized for minimal power consumption while meeting the target accuracy, it stores a combination of optimal formats for all layers. Then, it increases the data width DW by 1 bit for all layers and repeats the above procedure to search for other optimal formats, which can offer a trade-off between accuracy and area/power consumption. The above procedure is repeated until the DW reaches maximum data width (MAX DW), which is set to 32 bits in our experiment. Once the above search procedure is completed, the final step compares the accuracy and power of all search results and determines the best combination of formats with minimum power while maintaining the target accuracy.

## 5. Results and Analysis

### 5.1. Comparison of Floating-Point Arithmetic Operators 

The comparison of different formats is evaluated using the Design Compiler provided by Synopsys, which can synthesize HDL design to digital circuits for SoC. We have used TSMC 55nm process technology and a fixed frequency of 100 Mhz for our evaluation purpose. Table 4 shows the comparison of the synthesis results of floating-point adders/subtractors of various bit widths. Since there is just a difference of NOT gate between adder and subtractor, the adder/subtractor is considered as one circuit. 

It can be observed that the fewer the bits used for the floating-point adder/subtracter operation, the smaller the area or energy consumption. The floating-point format of each bit width is represented by *N* (*S*, *E*, *M*), where N indicates the total number of bits, S a sign bit, E an exponent, and M a Mantissa. 

The comparison of multipliers using various bit widths of floating-point formats is shown in Table 5.

As shown in Table 5, the fewer the bits used in floating-point multiplication, the smaller the area and energy consumption. The prominent observation in the multiplier circuit is that, unlike adder/subtractor, the energy consumption increases drastically. Finally, the comparison of division operators using various floating-point formats is shown in Table 6.

Although the operation delay time is constant compared to other operators, it can be seen that the smaller the number of bits used for the floating-point division operation, the smaller the area or energy consumption.

### 5.2. Evaluation of the Proposed CNN Training Accelerator

**Figure 11:**
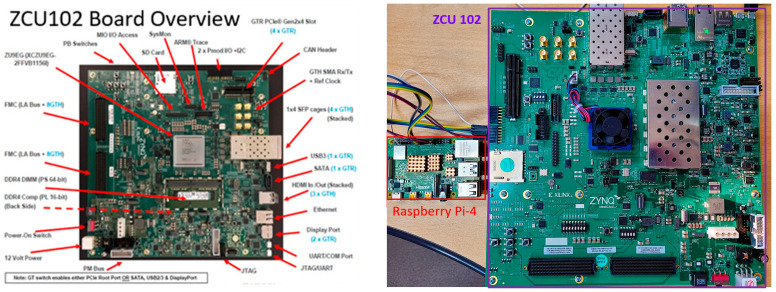
Hardware validation platform (FPGA ZCU102 and Host CPU Board).

We have implemented many test cases to determine the optimal arithmetic architecture without significantly compromising the accuracy of CNN. The proposed CNN training accelerator has been implemented in the register-transfer-level design using Verilog and verified using Vivado Verilog Simulator. After confirming the results, we implemented the accelerator on FPGA and trained all the test case models for 50K images. After training, the inference accuracy was calculated by providing 10K test images from the MNIST dataset to our trained models. Figure 11 shows the hardware validation platform. The images/weights, and control signals are provided to the FPGA board by the Host CPU board (Raspberry Pi) via the SPI interface.

Table 7 shows a few prominent format combinations—search results found by the proposed optimization algorithm of Fig. 11. Among these format combinations, Conv mixed-24 is selected as the most optimal format combination in terms of accuracy and power. This format combination uses a 24-bit format in the convolutional layer (forward and backpropagation), while assigning a 16-bit format for the Pooling, FC1, FC2, and SoftMax layers (forward- and backpropagation). 

**Figure 12:**
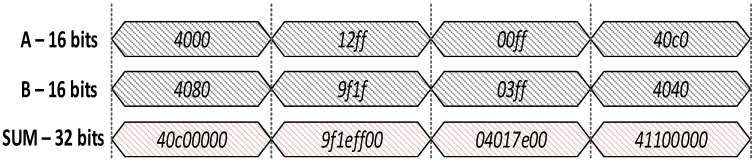
16-bit Adder with output in 32 bits.

As shown in Figure 12, the accumulation result for smaller numbers in the 16-bit convolution block’s adder can be displayed in 24 bits precisely and any bit exceeding 24 is redundant and does not improve the model accuracy. Therefore, in Conv mixed-24 precision, we used input image, input filters in 24-bit precision, and then performed the convolution operation. After that, we truncated the results to 16 bits for further processing. During backward convolution operation after 16-bit dot product operation, the accumulation is performed in 24 bits before updating the weights in the convolution block.

The results of FC1 Mixed-32 and FC2 Mixed-32 testify to the fact that since more than 90% of MAC operations are performed in the convolution layer, then increasing the precision of the convolution module has the highest impact on the overall accuracy. 

Table 8 compares our best architecture (Conv mixed-24) with existing works, which confirms that our architecture can substantially reduce hardware resources than the existing FGPA accelerators [28,33,34,35].

The energy consumption per image in the proposed accelerator is only 8.5 uJ, while it is 17.4 uJ in our previous accelerator [33]. Our energy per image is 1140, 81, and 555 times lower than the previous works [34], [28] and [35], respectively. 

## 6. Conclusions

This paper evaluated different floating-point formats and optimized the FP operators in the Convolutional Neural Network Training/Inference engine. It can operate on frequencies up to 80 Mhz, which increases the throughput of the circuit. It is 2.04-times more energy efficient, and it occupies a five times lesser area than its predecessors. We have used an MNIST handwritten dataset for our evaluation and achieved more than 93% accuracy using our mixed-precision architecture. Due to its compact size, low power, and high accuracy, our accelerator is suitable for AIoT applications. We will make our CNN accelerator flexible in the future to make the precision configurable on runtime. We will also add an 8-bit configuration in our flexible CNN accelerator to make it more compact and to reduce the energy consumption even more.

## Figures and Tables

**Table 1:** Formats evaluated in CNN.

Total Bits	Common Name	Significand Bits	Exponent Bits	Exponent Bias
16	Custom	10	6	$2^{5}-1=31$
16	Brain Floating	8	8	$2^{7}-1=127$
24	Custom	16	8	$2^{7}-1=127$
32	Single-Precision	24	8	$2^{7}-1=127$

**Table 2:** Comparison of division calculation using reciprocal and Signed Array.

Clock Frequency	50 Mhz		100 Mhz	
	Reciprocal	Signed Array	Reciprocal	Signed Array
Area (µm^2^)	38,018.24	6253.19	38,039.84	8254.21
Processing delay (ns)	70.38	21.36	64.23	10.79
Total Energy (pJ) ^a^	78.505	4.486	112.927	5.019

^a^ Total energy is an energy per division operation.

**Table 3:** Comparison of accuracy and dynamic power for different precisions.

S. No	Precision Format	Formats for Individual Layers	Mantissa	Exponents	Training Accuracy	Test Accuracy	Dynamic Power
1	IEEE-32	All 32-bits	24	8	96.42%	96.18%	36 mW
2	Custom-24	All 24-bits	16	8	94.26%	93.15%	30 mW
3	IEEE-16	All 16-bits	11	5	12.78%	11.30%	19 mW

**Table 4:** Comparison of N-bit floating-point adder/subtracter.

N-Bits	Common Name	Area (µm^2^)	Processing Delay (ns)	Total Energy (pJ)
16 (1,8,7)	Brain Floating	1749.96	10.79	0.402
24 (1,8,15)	Custom	2610.44	10.80	0.635
32 (1,8,23)	Single-Precision	3895.16	10.75	1.023

**Table 5:** Comparison of N-bit floating point multiplier.

N-Bits	Common Name	Area (µm^2^)	Processing Delay (ns)	Total Energy (pJ)
16 (1,8,7)	Brain Floating	1989.32	10.80	0.8751
24 (1,8,15)	Custom	2963.16	10.74	1.5766
32 (1,8,23)	Single-Precision	5958.07	10.76	3.3998

**Table 6:** Comparison of N-bit floating-point divider.

N-Bits	Common Name	Area (µm^2^)	Processing Delay (ns)	Total Energy (pJ)
16 (1,8,7)	Brain Floating	1442.16	10.80	0.6236
24 (1,8,15)	Custom	3624.12	10.79	1.9125
32 (1,8,23)	Single-Precision	8254.21	10.85	5.019

**Table 7:** Comparison of accuracy and dynamic power using the algorithm.

S. No	Precision Format	Formats for Individual Layers	Mantissa Bits	Exponent Bits	Training Accuracy	Test Accuracy	Dynamic Power
1	IEEE-16	All 16-bits	11	5	11.52%	10.24%	19 mW
2	Custom-16	All 16-bits	10	6	15.78%	13.40%	19 mW
3	Custom-16	All 16-bits	9	7	45.72%	32.54%	19 mW
4	Brain-16	All 16-bits	8	8	91.85%	90.73%	20 mW
5	CONV Mixed-18	Conv/BackConv-18 Rest 16-bits ^a^	10/8	8	92.16%	91.29%	21 mW
6	CONV Mixed-20	Conv/BackConv-20 Rest 16-bits ^a^	12/8	8	92.48%	91.86%	22 mW
7	CONV Mixed-23	Conv/BackConv-23 Rest 16-bits ^a^	15/8	8	92.91%	92.75%	22 mW
8	CONV Mixed-24	Conv/BackConv-24 Rest 16-bits ^a^	16/8	8	93.32%	93.12%	23 mW
9	FC1 Mixed-32	FC1/BackFC1-32 Rest 20-bits ^b^	24/12	8	93.01%	92.53%	26 mW
10	FC2 Mixed-32	FC1/BackFC1-32 Rest 22-bits ^c^	24/14	8	93.14%	92.71%	27 mW

^a^ Rest 16-bit modules are Pooling, FC1, FC2, Softmax, Back FC1, Back FC2 and Back Pooling. ^b^ Rest 20-bit modules are Convolution, Pooling, FC2, Softmax, Back FC2, Back Pooling and Back Conv. ^c^ Rest 16-bit modules are Convolution, Pooling, FC1, Softmax, Back FC1, Back Pooling and Back Conv.

**Table 8:** Comparison with other related work.

Criteria	[34]	[28]	[35]	[33]	Proposed
Precision	FP 32	FP 32	Fixed Point 16	FP 32	Mixed
Training dataset	MNIST	MNIST	MNIST	MNIST	MNIST
Device	Maxeler MPC-X	Artix 7	Spartan-6 LX150	Xilinx XCZU7EV	XILINX XCZU9EG
Accuracy	-	90%	92%	96%	93.32%
LUT	69,510	7986	-	169,143	33,404
FF	87,580	3297	-	219,372	61,532
DSP	23	199	-	12	0
BRAM	510	8	200	304	7.5
Operations (OPs)	14,149,798	-	16,780,000	114,824	114,824
Time Per Image (µs)	355	58	236	26.17	13.398
Power (W)	27.3	12	20	0.67	0.635 ^a^
Energy Per Image (µJ)	9691.5	696	4720	17.4	8.5077

^a^ Calculated by Xilinx Vivado (Power = Static power + Dynamic power).

## Data Availability

Not applicable.
